# Short-Chain Fatty Acids Differentially Affect Intracellular Lipolysis in a Human White Adipocyte Model

**DOI:** 10.3389/fendo.2017.00372

**Published:** 2018-01-11

**Authors:** Johan W. E. Jocken, Manuel A. González Hernández, Nicole T. H. Hoebers, Christina M. van der Beek, Yvonne P. G. Essers, Ellen E. Blaak, Emanuel E. Canfora

**Affiliations:** ^1^Department of Human Biology, NUTRIM School of Nutrition and Translational Research in Metabolism, Maastricht University Medical Centre+, Maastricht, Netherlands; ^2^Top Institute Food and Nutrition, Wageningen, Netherlands; ^3^Department of Surgery, NUTRIM School of Nutrition and Translational Research in Metabolism, Maastricht University Medical Centre+, Maastricht, Netherlands

**Keywords:** acetate, gut microbiota, adipose tissue, obesity, fat metabolism, hormone-sensitive lipase

## Abstract

**Background and aims:**

Gut-derived short-chain fatty acids (SCFA), formed by microbial fermentation of dietary fibers, are believed to be involved in the etiology of obesity and diabetes. Previous data from our group showed that colonic infusions of physiologically relevant SCFA mixtures attenuated whole-body lipolysis in overweight men. To further study potential mechanisms involved in the antilipolytic properties of SCFA, we aimed to investigate the *in vitro* effects of SCFA incubations on intracellular lipolysis and signaling using a human white adipocyte model, the human multipotent adipose tissue-derived stem (hMADS) cells.

**Methods:**

hMADS adipocytes were incubated with mixtures of acetate, propionate, and butyrate or single SCFA (acetate, propionate and butyrate) in concentrations ranging between 1 µmol/L and 1 mmol/L. Glycerol release and lipase activation was investigated during basal conditions and following β-adrenergic stimulation.

**Results:**

SCFA mixtures high in acetate and propionate decreased basal glycerol release, when compared to control (*P* < 0.05), while mixtures high in butyrate had no effect. Also, β-adrenergic receptor mediated glycerol release was not significantly altered following incubation with SCFA mixtures. Incubation with only acetate decreased basal (1 µmol/L) and β-adrenergically (1 µmol/L and 1 mmol/L) mediated glycerol release when compared with control (*P* < 0.05). In contrast, butyrate (1 µmol/L) slightly increased basal and β-adrenergically mediated glycerol release compared with control (*P* < 0.05), while propionate had no effect on lipolysis. The antilipolytic effect of acetate was accompanied by a reduced phosphorylation of hormone-sensitive lipase (HSL) at serine residue 650. In addition, inhibition of Gi G proteins following pertussis toxin treatment prevented the antilipolytic effect of acetate.

**Conclusion:**

The present data demonstrated that acetate was mainly responsible for the antilipolytic effects of SCFA and acts *via* attenuation of HSL phosphorylation in a Gi-coupled manner in hMADS adipocytes. Therefore, the modulation of colonic and circulating acetate may be an important target to modulate human adipose tissue lipid metabolism.

## Introduction

Increasing evidence suggests that the human gut microbiota and its products are key players in host metabolism, body weight, and insulin sensitivity, thereby contributing to the etiology of obesity and related disorders (1). The gut microbiota can ferment indigestible nutrients into short-chain fatty acids (SCFA), of which acetate, propionate and butyrate are the most abundant (2). Of note, these SCFA can be taken up by the epithelial lining of the gut and released into the blood stream (3). They may thereby act as important signaling molecules between gut microbiota and host physiology, by exerting effects on energy and substrate metabolism such as on adipogenesis and lipolysis in the adipose tissue (4).

Disturbances in adipose tissue function, characterized by a reduced capacity to store lipids, seem to play a major role in the development of insulin resistance and type 2 diabetes mellitus in humans (5). Under normal healthy conditions, the adipose tissue is an important buffering organ for daily postprandial fatty acid (FA) fluxes when endogenous lipolysis is inhibited. The adipose tissue thereby prevents excessive supply of lipids to nonadipose tissues such as liver, skeletal muscle, and pancreas. This buffering action may be impaired under obese insulin-resistant conditions (6, 7), resulting in increased circulating lipids and ectopic fat storage in nonadipose tissues, thereby provoking disturbances in insulin signaling and substrate metabolism (8, 9).

SCFA may affect adipose tissue lipid buffering capacity by affecting intracellular lipolysis, the process of hydrolysis of stored triacylglycerol into one molecule of glycerol and three FA molecules, and may thereby affect circulating lipid concentrations (4). Indeed, already decades ago, a decrease in plasma free fatty acids (FFA) after a single oral acetate ingestion was observed, pointing to an antilipolytic role of acetate (10). The more recent identification of the two pertussis toxin (PTX)-sensitive inhibitory G (Gi) protein-coupled receptors (GPRs) for SCFA, free fatty acid receptor 3 (FFAR3, also known as GPR41), and FFAR2 (also known as GPR43) in human adipose tissue (11), has led to renewed interest in the lipolytic properties of SCFA. In addition, a direct association between the SCFA/FFAR signaling pathway and lipolytic activity in murine adipocytes was recently discovered (12). Treatment of differentiated murine 3T3-L1 adipocytes with acetate and propionate in a range between 0.1 and 0.3 mmol/L exhibited FFAR2 activation and a reduction in intracellular lipolytic activity as assessed by a decreased release of glycerol in the culture medium (12). In contrast, incubation of 3T3-L1 adipocytes with supraphysiological concentrations of propionate (20 mmol/L) or butyrate (5 mmol/L) resulted in enhanced glycerol release (13). However, in murine 3T3-L1 adipocytes only FFAR2, and not FFAR3, is expressed (14–17). Therefore, it remains to be determined, whether these findings extend to human adipocytes, in which both FFAR3 and FFAR2 are expressed (11, 18). Thus, further investigation on the role of SCFA in human adipocyte lipolysis is urgently warranted.

We recently observed that colonic infusions of mixtures of acetate, propionate and butyrate, in ratios and concentrations that can be reached after dietary fiber intake, attenuated whole-body lipolysis in overweight normoglycaemic men (19). Therefore, the aim of the present study was to elucidate whether an altered intracellular adipocyte lipolytic rate is responsible for the antilipolytic effect of SCFA found *in vivo*, as well as to further investigate underlying mechanisms. Hence, we investigated the *in vitro* effects of incubation with SCFA mixtures and single SCFA on intracellular lipolysis in a human white adipocyte model, the human multipotent adipose tissue-derived stem (hMADS) cells. To study whether these effects are mediated *via* Gi-coupled receptors, we investigated the effect of SCFA on lipase activation and performed PTX-mediated inhibition of FFARs.

## Materials and Methods

### Cell Culture

Human multipotent adipose tissue-derived stem cells, a validated human white adipocyte model to study lipid metabolism (20), were obtained from human subcutaneous adipose tissue biopsies and differentiated into the adipogenic lineage. As described previously by Jocken et al. (21), cells were seeded at a density of 2,000 cells/cm^2^ and kept in proliferation medium [Dulbecco’s modified Eagle’s medium (DMEM) and Ham’s F-12 Nutrient Mixture (Gibco, Bleiswijk, Netherlands), 10% fetal bovine serum (Bodinco BV, Alkmaar, NL, Netherlands), and 50 U/ml penicillin (Gibco), 50 µg/ml of streptomycin (Gibco)]. At 70–80% confluence 250 µmol/L IBMX (Sigma, St. Louis, MI, USA) and 5 µmol/L rosiglitazone (Enzo Life Sciences, Raamsdonksveer, Netherlands) were added to induce adipogenic differentiation. The lipolytic experiments were carried out between days 12 and 14 of the differentiation.

Pooled hMADS cells were derived from male human donors with a large range in BMI (20–40 kg/m^2^) and glucometabolic status. The male donors were aged between 35 and 70 years and participated in two different clinical trials performed (http://ClinicalTrials.gov, NCT02241421 and NCT02598544). The study protocols were approved by the Medical Ethical Committee Jessa hospital, Hasselt and Hasselt University, Belgium, and by the Medical Ethical Committee of Maastricht University Medical Center, Maastricht, The Netherlands. All procedures were according to the declaration of Helsinki (revised version, October 2008).

### Lipolysis Experiment

#### Free Glycerol Release Analysis

To study effects of SCFA mixtures on the basal glycerol release, hMADS adipocytes were incubated for 6 h with 300 µL DMEM 3% fatty-acid free BSA (Sigma-Aldrich, St. Louis, MI, USA) supplemented with or without a mixture high in acetate containing 80% acetate, 10% propionate and 10% butyrate (80:10:10), a SCFA mixture containing 60% acetate, 20% propionate and 20% butyrate (60:20:20), a mixture high in PA containing 40% acetate, 35% propionate and 25% butyrate (40:35:25) and a mixture high in BA containing 40% acetate, 25% propionate, and 35% butyrate (40:25:35) in final concentrations of 1 mmol/L or 1 µmol/L for 6 h.

To study effects of single SCFA on the basal (non-stimulated) glycerol release, hMADS adipocytes were incubated with 300 µL DMEM 3% fatty-acid free BSA (Sigma-Aldrich, St. Louis, MI, USA) supplemented with or without acetate (Merck, Darmstadt, Germany), propionate (Sigma-Aldrich, St. Louis, MI, USA), or butyrate (Sigma-Aldrich, St. Louis, MI, USA) at a final concentration of 1 mmol/L and 1 µmol/L concentrations for 6 h.

To examine the effects of single SCFA or SCFA mixtures on the β-adrenergic receptor-mediated glycerol release, 30 min after the initiation of SCFA incubation the non-selective β-agonist isoprenaline (ISO) was added at a final concentration of 1 µmol/L.

After 6 h incubation, the plates were placed on ice to stop the reactions, and subsequently 250 µL supernatant was removed and directly snap-frozen in liquid nitrogen and stored at −80°C until analysis. The glycerol concentrations were quantified using a commercial fluorometric assay (EnzyChrome™ Adipolysis assay kit, BioAssay Systems, Hayward, CA, USA).

#### Gene Expression of FFAR3 and FFAR2 in hMADS Adipocytes

To determine the FFAR3 and FFAR2 mRNA expression, total RNA was extracted from hMADS adipocytes at days 0, 2, 7, 10, and 12 using TRIzol reagent (Invitrogen) and SYBR-Green based real-time PCRs were performed using an iCylcer (Biolegio, Nijmegen, The Netherlands; primer sequence see Table 1). Results were normalized for 18S ribosomal RNA (calculating delta-delta Ct values). Ct values ranged from 27 to 33 for FFAR3/2 and 6 to 9 for 18S ribosomal RNA.

**Table 1:** Primer sequences.

Gene	Forward (5′→3′)	Reverse (3′→5′)
18S	AGTTAGCATGCCAGAGTCTCG	TGCATGGCCGTTCTTAGTTG
FFAR3 (GPR41)	TTCACCACCATCTATCTCACCG	GGAACTCCAGGTAGCAGGTC
FFAR2 (GPR43)	CCGTGCAGTACAAGCTCTCC	CTGCTCAGTCGTGTTCAAGTATT

*FFAR, free fatty acid receptor; GPR, G-protein-coupled receptor*.

#### Western Blotting

To study the effects of acetate on the protein expression and activation (phosphorylation) of the key lipolytic enzymes adipose triglyceride lipase (ATGL) and hormone-sensitive lipase (HSL), hMADS adipocytes were incubated with 300 µL DMEM 3% BSA supplemented with or without acetate at a final concentration of 1 µmol/L for 1 h. In addition, 30 min after the start of the acetate incubation, ISO was added to the medium at a final concentration of 1 µmol/L to investigate the effects of acetate on the β-adrenergic receptor mediated HSL phosphorylation. Following 1-h incubation, cells were washed twice with ice-cold phosphate-buffered saline, and cells were homogenized in radioimmunoprecipitation assay buffer supplemented with a protease and phosphatase inhibitor cocktail (Cell Signaling, Leiden, The Netherlands). 20 µg solubilized proteins were separated on a Criterion TGX precast gel (Bio-Rad), transferred using the Trans Blot Turbo transfer system (Bio-Rad), and incubated with primary antibodies. The HSL antibody was a kind gift from Prof. C. Holm (Lund University, Lund, Sweden). The ATGL (No.: 2138) and the phosphorylated HSL (pHSL) at serine residue 650 (rat SER660) (No.: 4126) antibodies were both obtained from Cell Signaling Technology, Leiden, The Netherlands. To determine the FFAR3 (antibody No.: 103718, Abcam, Cambridge, UK) and FFAR2 (antibody No.: 131003, Abcam, Cambridge, UK) protein expression, total protein was extracted from hMADS adipocytes at days 0, 2, 4, 7, 9, 11, and 14.

#### Effect of Inhibition of Gi Proteins Using PTX

To study the putative involvement of the Gi-type G-protein-coupled FFARs in the acetate-induced inhibition of the lipolytic response, we incubated hMADS adipocytes for 6 h with acetate in a concentration of 1 µmol/L during basal and ISO stimulated conditions (see protocol above), with or without supplementation of PTX for the whole 6 h to the medium at a final concentration of 100 ng/mL (No.: P7208, Sigma-Aldrich, St. Louis, MI, USA). The PTX experiments were conducted according the Guidelines of the European Chemicals Agency (Helsinki, Finland).

### Statistical Analysis

Values are expressed as mean ± SD. Significance was determined using the nonparametric Mann Whitney U-test when comparing two groups (single SCFA, Western blotting, and PTX experiment) or the Kruskal–Wallis *H*-test when comparing more groups (SCFA mixtures). In case of significant Kruskal–Wallis *H*-test, Dunns *post hoc* test was performed. Statistics were performed using the GraphPad Prism 5.0a software package (GraphPad Software, San Diego, CA, USA). and *P* < 0.05 (two-sided *P*-value) was considered statistically significant.

## Results

### SCFA Mixtures High in Acetate and Propionate Decrease Basal Adipocyte Glycerol Release *In Vitro*

**Figure 1:**
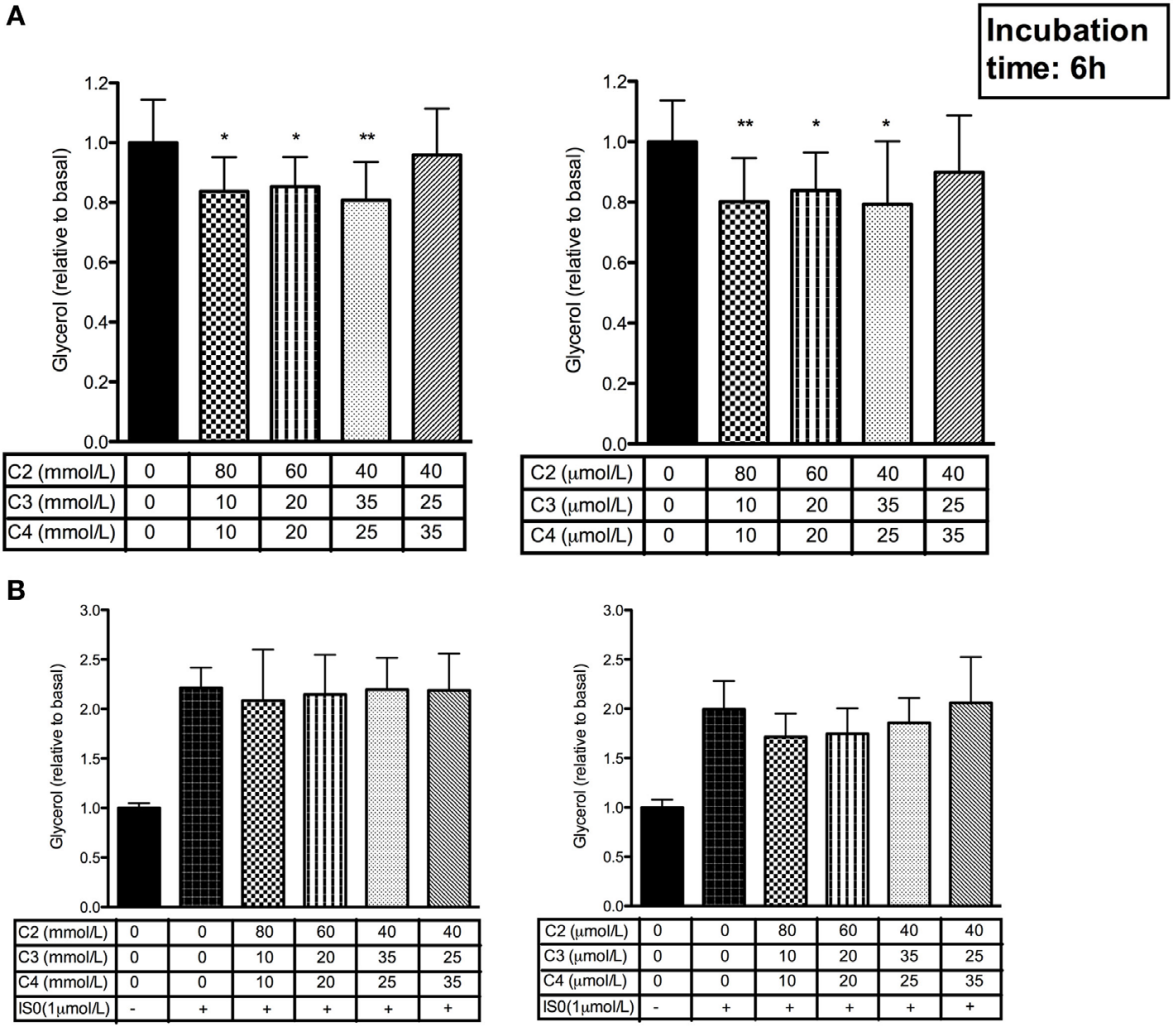
Effect of short-chain fatty acid (SCFA) mixtures on basal and β-adrenergic receptor stimulated glycerol release in human multipotent adipose tissue-derived stem adipocytes. **(A)** Basal (non-stimulated) glycerol concentrations during 6 h incubation with 1 mmol/L or 1 µmol/L SCFA mixtures including acetate (C2), propionate (C3), and butyrate (C4). **(B)** Effect of 6 h incubation with 1 mmol/L or 1 µmol/L SCFA mixtures including acetate (C2), propionate (C3), and butyrate (C4) on β-adrenergic receptor stimulated (1 µmol/L isoprenaline) glycerol release; Values are given as means ± SD (*n* = 4–6 independent experiments). Statistical significance compared to basal indicated as asterisk (*) when *P* < 0.05 and double asterisk (**) when *P* < 0.01.

We investigated whether the observed decrease in systemic glycerol found in our *in vivo* study (19) is related to an attenuated adipocyte intracellular lipolysis. Therefore, we incubated hMADS adipocytes for 6 h with 1 µmol/L and 1 mmol/L of SCFA mixtures. The SCFA mixtures high in acetate and propionate (80:10:10, 60:20:20, and 40:35:25) decreased the basal (non-stimulated) glycerol release, when compared with control (*P* < 0.05, Figure 1A). The SCFA mixture high in butyrate (40:25:35) did not significantly affect basal glycerol release (Figure 1A). In contrast to the decreased basal lipolysis, β-adrenergic receptor mediated glycerol release was not significantly affected following incubation with all SCFA mixtures in physiological concentrations ranging from 1 µmol/L to 1 mmol/L (Figure 1B).

### Single SCFA Differentially Affect Adipocyte Glycerol Release *In Vitro*

**Figure 2:**
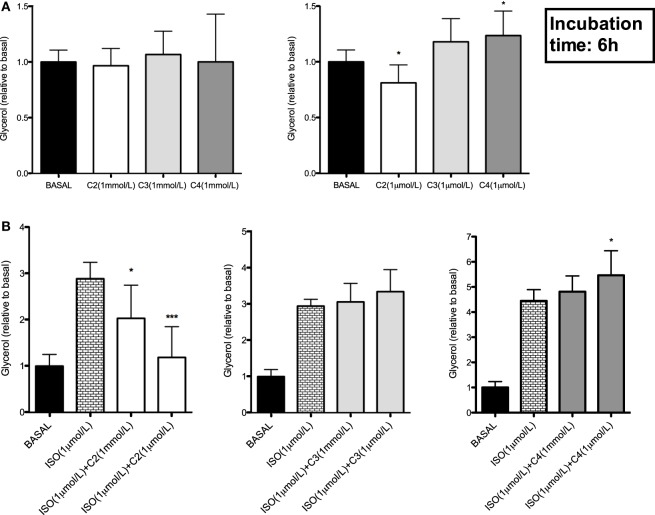
Effect of single short-chain fatty acid on basal and β-adrenergic receptor stimulated glycerol release in human multipotent adipose tissue-derived stem adipocytes. **(A)** Basal (non-stimulated) glycerol concentrations during 6 h incubation with 1 mmol/L or 1 µmol/L acetate (C2), propionate (C3), or butyrate (C4). **(B)** Effect of 6 h incubation with 1 mmol/L or 1 µmol/L acetate (C2), propionate (C3), or butyrate (C4) on β-adrenergic receptor stimulated (1 µmol/L isoprenaline) glycerol release; values are given as means ± SD (*n* = 4–7 independent experiments). Statistical significance when compared with basal indicated as asterisk (*) when *P* < 0.05 and as triple asterisk (***) when *P* < 0.001.

Subsequently, we studied whether one particular SCFA was responsible for the observed antilipolytic effect. Therefore, we incubated hMADS adipocytes for 6 h with acetate, propionate and butyrate in concentrations of 1 µmol/L and 1 mmol/L. Incubation with 1 µmol/L acetate decreased basal glycerol release when compared to control cells (*P* < 0.05, Figure 2A). In addition, acetate blunted the β-adrenergic receptor-mediated glycerol release in concentrations of 1 mmol/L and 1 µmol/L, when compared to control (*P* < 0.05, Figure 2B). In contrast, 1 µmol/L butyrate treatment slightly increased basal (*P* < 0.05, Figure 2A) and β-adrenergic receptor mediated glycerol release (*P* < 0.01, Figure 2B), when compared to control treated cells. Neither in the basal state nor during β-adrenergic receptor stimulation, a significant difference between propionate and control treated adipocytes were observed (Figures 2A,B).

### Acetate Attenuates HSL Phosphorylation in Adipocytes

**Figure 3:**
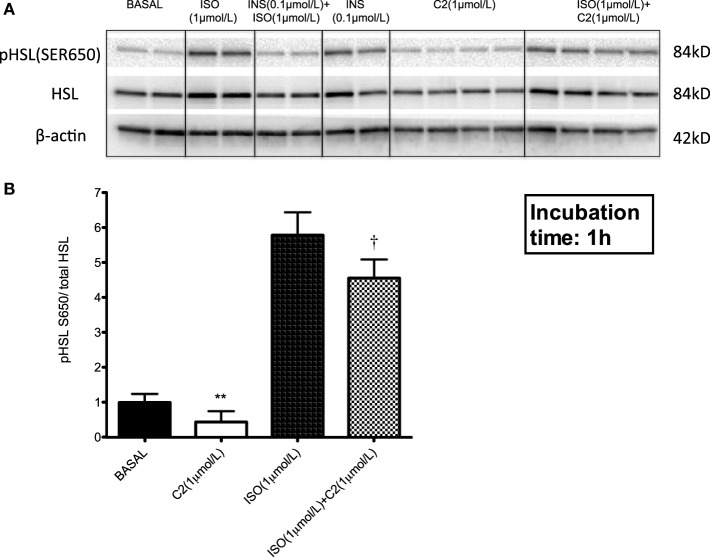
Acetate attenuates hormone-sensitive lipase (HSL) (SER 650) phosphorylation in human multipotent adipose tissue-derived stem adipocytes. **(A)** Representative Western blot showing that 1 µmol/L acetate (C2) reduced the relative amount of HSL phosphorylated on serine 650 in the presence of isoprenaline (ISO). In this blot, insulin was used as a control. See for corresponding entire blots in Figure S1 in Supplementary Material **(B)** Quantification of Western blot using ImageLab 3.0 normalized to total HSL (*n* = 4). Values are given as means ± SD. Statistical significance when compared with basal indicated as double asteriks (**) when *P* < 0.01; and when compared with ISO as dagger (^†^) when *P* < 0.05.

Since the above data indicated that mainly acetate is the driver of the antilipolytic effect of SCFA in human adipocytes, we subsequently investigated the underlying mechanisms in more detail by quantification of key enzymes involved in intracellular lipolysis, including ATGL, HSL, and pHSL. No differences of acetate on total HSL or ATGL protein content were observed (Figure 3A for total HSL protein). As indicated in Figure 3B, treatment of hMADS adipocytes with 1 µmol/L acetate resulted in a reduction in the relative amount of phosphorylation of HSL on the serine 650 when compared with control non-treated cells (*P* < 0.01, Figure 3B). As expected, the phosphorylation of HSL on the serine 650 increased by fivefold to sixfold in the presence of ISO, when compared with non-stimulated adipocytes (Figures 3A,B). However, pre-treatment of hMADS adipocytes with 1 µmol/L acetate and ISO resulted in reduction in the relative amount of pHSL_(SER650)_ compared to ISO stimulation alone (*P* < 0.05, Figure 3B).

### PTX Treatment Prevents the Antilipolytic Effect of Acetate in hMADS Cells

**Figure 4:**
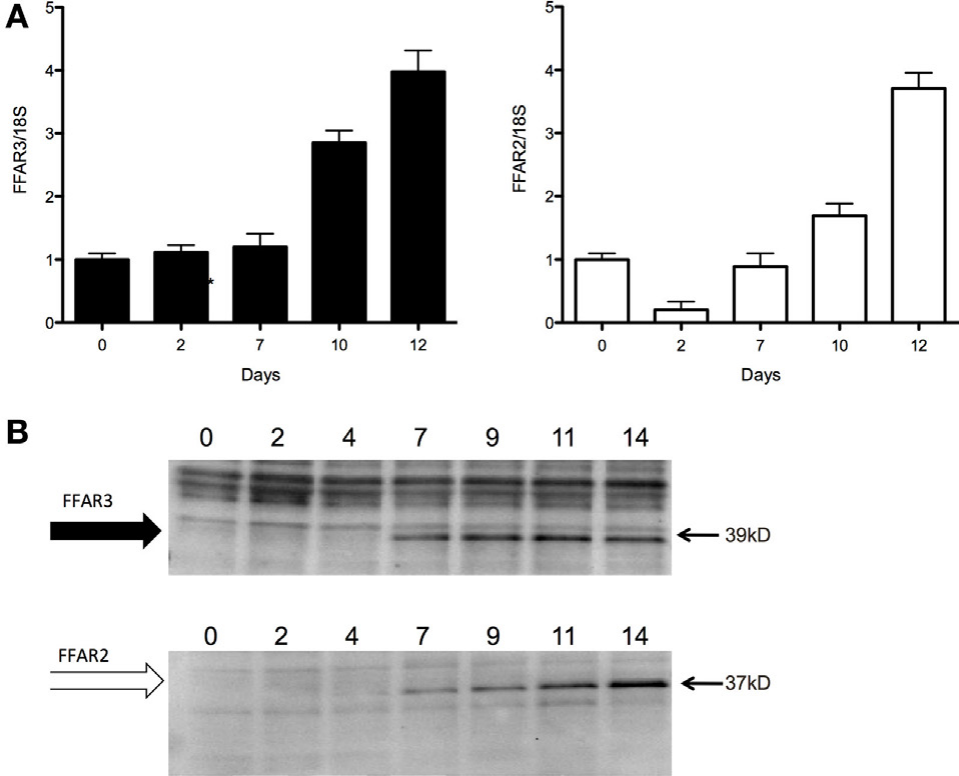
Free fatty acid receptor (FFAR) 3 and FFAR2 are expressed at the RNA and protein level in human multipotent adipose tissue-derived stem adipocytes. **(A)** FFAR3/2 mRNA expression during adipocyte differentiation (days 0–12). **(B)** FFAR3/2 protein expression during adipocyte differentiation (days 0–14) (*n* = 1), See for corresponding entire blots in Figure S2 in Supplementary Material.

Finally, we investigated the involvement of inhibitory Gi G-protein-coupled receptors in this acetate-mediated antilipolytic effect. Both FFAR3 and FFAR2, the major SCFA receptors, were expressed at the RNA (Figure 4A) and protein (Figure 4B) level in our hMADS cells, and expression increased during adipogenic differentiation with a maximal expression at days 12 and 14 (see Figure 4; Figure S3C in Supplementary Material for FFAR2 protein expression in hMADS at day 14).

**Figure 5:**
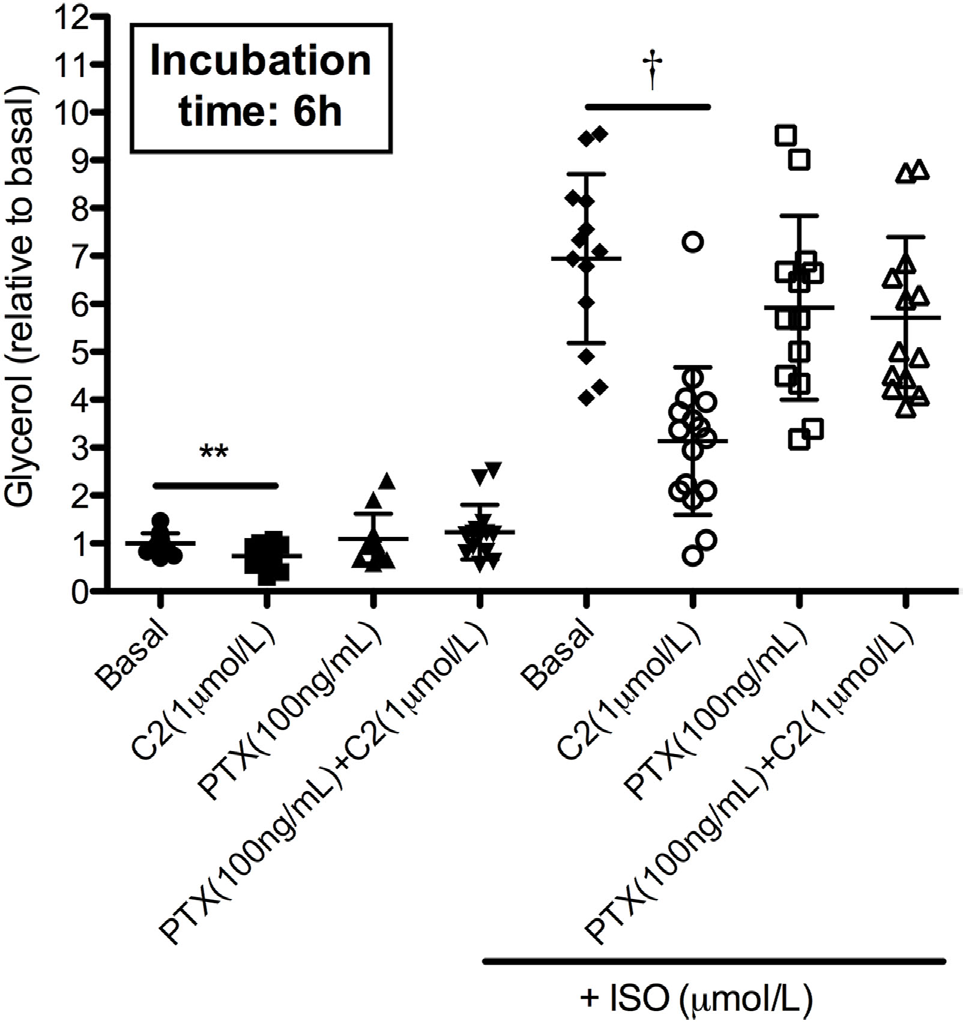
Pertussis toxin (PTX) abrogated acetate-induced inhibition (1 µmol/L) of isoprenaline (ISO)-mediated glycerol release in human multipotent adipose tissue-derived stem (hMADS) adipocytes. Values are given as individual points and means ± SD (*n* = 4 independent experiments). Statistical significance when compared to basal indicated as asterisk (*) when *P* < 0.01; and when compared with ISO as dagger (^†^) when *P* < 0.001.

Next, hMADS adipocytes were incubated with or without PTX, which irreversibly blocks Gi function, thereby inhibiting both FFAR3 and FFAR2 in our hMADS adipocytes. Of interest, PTX prevented the acetate-mediated (1 µmol/L) decrease in basal and β-adrenergic receptor stimulated glycerol release (*P* < 0.01, Figure 5).

## Discussion

**Figure 6:**
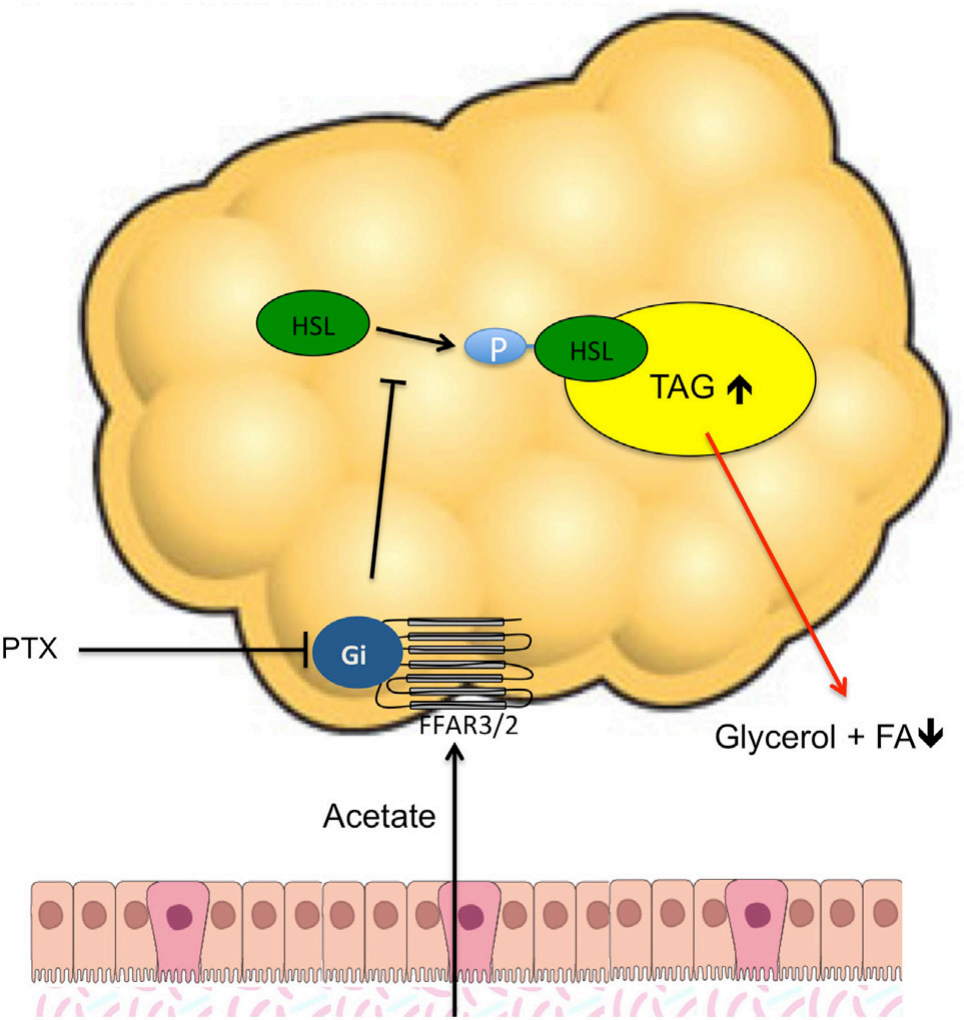
Proposed mechanism of the acetate-mediated antilipolytic effect in human adipocytes. The blunted fatty acid and glycerol release during acetate incubation is accompanied by a reduced phosphorylation of HSL_(SER650)_, indicating a role for protein kinase A in this antilipolytic process The free fatty acid receptor (FFAR) inhibitor pertussis toxin prevents the acetate–mediated antilipolytic effect, indicating a role for a Gi protein-coupled receptor mechanism (i.e., FFAR3 and/or FFAR2) in human adipocyte lipolysis.

This study provides new insight in the effects of SCFA on human adipocyte lipolysis. We previously showed that acute colonic administration of three physiological-relevant SCFA mixtures, and subsequent elevated circulating acetate concentrations, reduced circulating glycerol concentration in overweight males, indicative of a reduced whole-body lipolysis (19). However, to further investigate whether the reduction in whole-body lipolysis was related to a putative SCFA effect on white adipocyte intracellular lipolysis, we performed several *in vitro* experiments using our validated hMADS adipocyte model. Our present *in vitro* study in hMADS adipocytes demonstrated that mainly acetate had antilipolytic effects, which was accompanied by a reduced phosphorylation of HSL (at SER650). Incubation with the Gi inhibitor PTX prevented the acetate-mediated antilipolytic effect, suggesting that this antilipolytic effect may be mediated through an acetate-FFAR-coupled signaling pathway (for an schematic overview see Figure 6).

This study demonstrated that SCFA mixtures at physiological (1 µmol/L) and more supraphysiological (1 mmol/L) concentrations attenuate intracellular lipolysis in human adipocytes. Furthermore, by subsequently incubating human adipocytes with single SCFA, we demonstrated that the intestinally and systemically most abundant SCFA acetate seems to be the main driver of this antilipolytic effect. Acetate is the most abundant circulating SCFA and is found in serum and plasma mean concentrations varying from 5 up to 220 µmol/L, depending on the nutritional status (3, 22–24). Propionate and butyrate are found at much lower maximal mean concentrations of 13 and 12 µmol/L, respectively (3, 22, 23). However, no human data are available reflecting SCFA concentrations that reach the adipose tissue *via* their capillaries. Based on the scarcely available data on circulating SCFA concentrations, an acetate to propionate to butyrate ratio of 80:10:10 and 60:20:20 could resemble physiologically circulating concentrations and the SCFA concentrations used in this study might be in a physiological (1 µmol/L) to supraphysiological (1 mmol/L) range. Interestingly, the most pronounced effects on lipolysis were found with an acetate concentration of 1 µmol/L, which thus seems to be lower than circulating concentrations. Therefore it would be of major interest to measure actual acetate concentrations in adipose tissue capillaries or interstitial fluids. To the knowledge of the authors, no data on this are available, which would be very interesting to detect *via* for example microdialysis techniques.

In addition, we observed that the antilipolytic effect of acetate was accompanied by a reduced phosphorylation of HSL at the serine 650, a major protein kinase A (PKA) regulatory site. In accordance with this observation, Aberdein et al. (25) indicated that treatment of murine 3T3-L1 adipocytes with supraphysiological concentrations (4 mmol/L) of sodium acetate reduced the β-adrenergic receptor stimulated non-esterified FA release and decreased HSL phosphorylation at another PKA regulatory site (serine 563) (25). Ge et al. (12) showed that treatment of 3T3-L1 adipocytes with acetate and propionate in a range between 0.1 and 0.3 mmol/L reduced the basal and β-adrenergic receptor stimulated intracellular lipolytic activity as assessed by a decreased release of glycerol in the culture medium (12). In contrast to the antilipolytic effect of acetate, we observed a slightly increased basal lipolytic response after butyrate treatment in hMADS adipocytes. Comparable results were reported by Rumberger et al. (13) showing an increased basal lipolytic response (glycerol release) following incubation of murine 3T3-L1 adipocytes with 5 mmol/L butyrate (13). However, the underlying mechanism of this lipolytic effect of butyrate needs further investigation in hMADS cells. Together, these results suggest that acetate is the main driver for the antilipolytic effects of SCFA, which is accompanied by an attenuated HSL phosphorylation in both, murine and human adipocytes.

Finally, we observed that the antilipolytic effect of acetate might be FFAR dependent. We first showed in accordance to other reports in human adipocyte models (18) that both FFAR3 and FFAR2 transcripts and protein are expressed in our hMADS adipocyte model, and that both increased during adipogenic differentiation. Furthermore, we showed that the effects of acetate are abrogated with co-incubation of PTX. PTX is a well-known FFAR inhibitor and irreversible inactivates Gi proteins. Thereby, these data suggest that the acetate effects were mediated *via* a Gi protein receptor-PKA pathway. In line, a previous study in murine 3T3-L1 adipocytes has shown that the lipolytic effect of acetate was mediated by activation of FFAR2 (12). However, further investigations are warranted to elucidate whether SCFA effects on human intracellular adipocyte lipolysis are mediated mainly *via* FFAR2 and/or FFAR3, using specific human knockdown models. In particular the role of FFAR3/2 protein should be further investigated *via* the use of knockdown and overexpression in human adipocyte models.

Furthermore, evidence is increasing that metabolic phenotype should be considered in future lipolysis studies. Present literature provides evidence that obesity-related metabolic disturbances, such as insulin resistance, are linked to differences in circulating acetate levels and acetate-induced metabolic responses. For example, in our acute studies (19, 24) we included overweight and obese individuals with average fasting acetate concentrations of approximately 20–50 µmol/L, whereas in another study of our group with insulin resistant obese individuals markedly higher acetate concentrations of approximately 70–90 µmol/L have been found (26). In addition, a kinetic study showed that the acetate clearance rate is lower and the half-life is longer in type 2 diabetic patients when compared with healthy normoglycaemic controls (27). This suggests a disturbed uptake and/or metabolism of acetate, which might be relevant to elicit acetate-induced metabolic effects and cell signaling in peripheral tissues. Furthermore, there are indications that overweight insulin resistant compared to normoglycaemic individuals have lower acetate-induced antilipolytic responses on a whole-body level (28). An acute study demonstrated that intravenously administered acetate resulted in a greater FFA fall and rebound in healthy adults compared with hyperinsulinaemic individuals (28). Therefore, comparing SCFA-mediated inhibition of the lipolytic response and intracellular signaling mechanism in adipocytes derived from normoglycaemic, insulin sensitive versus metabolically more compromised donors is of major interest. Here, we included cells from human adults with a wide range of BMI and glucometabolic status, therefore we did not distinguish between metabolic phenotypes, which is as limitation.

Nevertheless, this study has clinical implications. If the observed results can be translated into long-term *in vivo* metabolic effects, increased systemic acetate availability might improve human white adipose tissue lipid buffering capacity and reduce adipose tissue lipid spillover. This could ultimately result in attenuated ectopic fat accumulation and improved insulin action in insulin sensitive tissues such as skeletal muscle, pancreas and liver, preventing insulin resistance. The present study showed that acetate induces a partial inhibition of intracellular lipolysis during basal conditions. Interestingly, combined data derived from rodents and humans demonstrated that a comparable partial inhibition of intracellular lipolysis has beneficial effects on insulin sensitivity without affecting adipose tissue mass in the longer term (29, 30). In addition to elevated basal lipolysis, β-adrenergic receptor agonist sensitivity is blunted in obese insulin resistant individuals (31–33), which poses the question whether a further decrease in β-adrenergically mediated lipolysis by SCFA is positive with respect to metabolic health. Thus further research including isoprenaline concentration–response curves are needed to ascertain SCFA-induced changes in efficiency or potency of β-adrenergic receptor agonists in different metabolic phenotypes.

With the present *in vitro* study using our human adipocyte model, we explored a mechanism that might explain the previously *in vivo* observed antilipolytic effect of physiologically relevant SCFA mixtures (19). However, other mechanisms, which might also contribute to the SCFA-induced antilipolytic effect on whole-body level, could not be excluded here. For example, in parallel to acetate concentrations, circulating peptide YY (PYY) concentrations were increased after the colonic infusions of SCFA mixtures in our *in vivo* experiment (19). Indeed, PYY was previously recognized for its antilipolytic property in human adipocytes (34). In addition, an intriguing study in rodents showed that SCFA can influence energy homeostasis including lipolysis *via* dorsal sympathetic ganglions and spinal pathways (35).

In conclusion, we demonstrated that in particular the colonic and peripheral most abundant SCFA acetate plays an important role in the regulation of human adipose tissue lipolysis. We showed that the luminal and systemically most abundant SCFA acetate was mainly responsible for the antilipolytic response, *via* FFAR-mediated attenuation of HSL phosphorylation in human adipocytes. Indicating that the modulation of colonic and systemic acetate might be a target to prevent or improve insulin resistance in human. Therefore, future studies should focus on increasing nutritional strategies to enhance circulating acetate availability, for example *via* supplementation of specific acetogenic fibers, to improve human lipid metabolism.

## Ethics Statement

hMADS cells, a validated human white adipocyte model to study lipid metabolism, were obtained from human subcutaneous adipose tissue biopsies from male donors. The male donors participated in two different clinical trials (http://ClinicalTrials.gov, NCT02241421 and NCT02598544). The study protocols were approved by the Medical Ethical Committee Jessa hospital, Hasselt and Hasselt University, Belgium, and by the Medical Ethical Committee of Maastricht University Medical Center, Maastricht, The Netherlands. All procedures were according to the declaration of Helsinki (revised version, October 2008).

## Author Contributions

JE, EB, and EC were responsible for the study concept and design, analysis and interpretation of the data, and critical revision of the manuscript for important intellectual content. MH, YE, and NH generated data. JE and EC acquired all data, completed statistical analysis, and drafted the manuscript. CB critically revised the manuscript. EB obtained funding and supervised the study.

## Conflict of Interest Statement

The authors declare that the research was conducted in the absence of any commercial or financial relationships that could be construed as a potential conflict of interest.
