# Supplementary material for: Short-Chain Fatty Acids Differentially Affect Intracellular Lipolysis in a Human White Adipocyte Model
Source: Front Endocrinol (Lausanne). 2018 Jan 11;8:372. doi: 10.3389/fendo.2017.00372 (PMC5768634; doi:10.3389/fendo.2017.00372)
Supplement: Supplementary file 1 [file Data_Sheet_1.PDF]

Supplementary Material

Short-chain fatty acids differentially affect intracellular lipolysis in a human white adipocyte model

Johan W.E. Jocken[1, 2], Manuel A. González Hernández[1], Nicole T.H. Hoebers[1], Christina M. van der Beek[2, 3], Yvonne P.G. Essers[1], Ellen E. Blaak[1, 2,] Emanuel E. Canfora[1, 2*]

[1] Department of Human Biology, NUTRIM 'School of Nutrition and Translational Research in Metabolism', Maastricht University Medical Centre+, 6229ER Maastricht, The Netherlands
[2] Top Institute Food and Nutrition, 6700AN Wageningen, the Netherlands
[3]Department of Surgery, NUTRIM 'School of Nutrition and Translational Research in Metabolism', Maastricht University Medical Centre+, 6229ER Maastricht, The Netherlands

Correspondence: Dr. Emanuel E. Canfora, Emanuel.canfora@maastrichtuniversity.nl

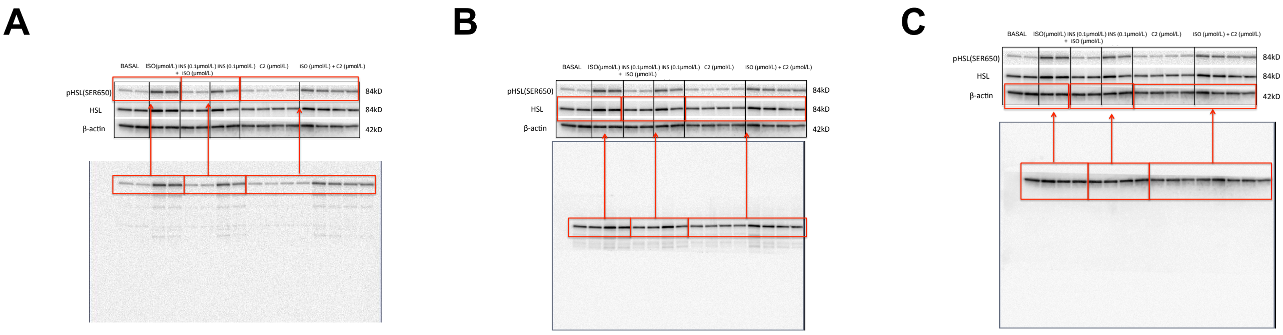

Supplementary figure 1: Corresponding blots for **figure 3A** *Acetate attenuates HSL (SER 650) phosphorylation in hMADS adipocytes* of the manuscript.

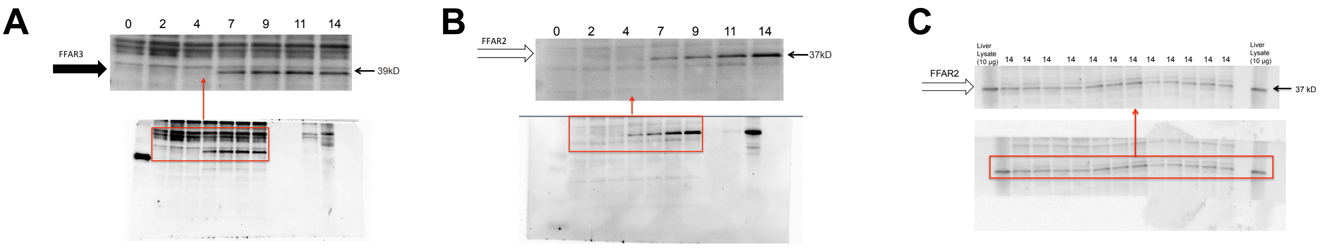

Supplementary figure 2: A and B: Corresponding blots for **figure 4B** *FFAR3 and FFAR2 are expressed at the protein level in hMADS adipocytes* of the manuscript **C:** Additional blot of FFAR2 protein expression of fully differentiated hMADS adipocytes at day 14 (as positive control 10 µg human fetal liver lysate was used).
